# Reality matters: in-field validation of fresh frozen cadaver-based airway training in HEMS - the BOAH Initiative: a prospective observational cohort study

**DOI:** 10.1186/s12873-026-01729-y

**Published:** 2026-08-26

**Authors:** Sebastian Imach, Tobias Ahnert, Sarah Krueger, Mai Bui, Bernd A. Leidel, Jenny Patricia Kehrberger, Michael Kemper, Benny Kölbel

**Affiliations:** 1 Department of Trauma and Orthopedic Surgery, Cologne-Merheim Medical Center (CMMC), University Witten/Herdecke, Cologne, Germany; 2 Department of Emergency Medicine, Hospital Mechernich, Mechernich, Germany and Medical Director Emergency Medical Services District Euskirchen, Mechernich, Germany; 3 Helicopter Emergency Medical Service (HEMS), Christoph 3, Cologne, Germany; 4 Department of Emergency Medicine, Campus Benjamin Franklin,Charité–Universitätsmedizin Berlin, Berlin, Germany; 5 Department of Emergency Medicine, Vivantes Auguste-Viktoria-Klinikum, Berlin, Germany; 6 Department of Anaesthesiology, University Hospital Aachen, Aachen, Germany; 7 Department of General, Visceral and Thoracic Surgery, Armed Forces Hospital Ulm, Ulm, Germany

**Keywords:** First-pass success rate endotracheal intubation, Difficult airway management, Airway management in critically ill patients, Airway management in trauma HALO procedures, Training in airway management, Medical simulation

## Abstract

**Background:**

Endotracheal intubation (ETI) remains the gold standard for emergency airway management. The performance indicator is the first-pass success rate (FPSR). Providers without routine practice in elective airway management need adapted training to gain proficiency and maintain a high level of competence. Fresh frozen cadavers (FFCs) have been shown to be effective in training and can be modified to recreate difficult airway features seen in helicopter emergency service (HEMS).

**Methods:**

In this prospective before-and-after study, the BASELINE phase consisted of recording both the performance of 16 HEMS physicians during 60 consecutive ETIs at Cologne HEMS and the corresponding airway characteristics of the patients. FPSR-raising measures for emergency airway management were identified through a structured literature review process and compiled into a bundle—the Best Of Airway management in HEMS (BOAH) bundle. The bundle was trained on five modified FFCs representing airway characteristics of the BASELINE phase. All the HEMS physicians and eight HEMS technical crew (HEMS-TC) members who were part of the BASELINE phase completed the training with videolaryngoscopy (VL) and a bougie-first strategy. Subsequently, another continuous 60 ETIs by the same teams were evaluated (BOAH phase). The primary outcome measure was the FPSR before and after the implementation of FFC training. Second, we evaluated the duration of ETIs and the oxygen saturation (SpO2) after ETIs.

**Results:**

In the BOAH phase, 61 of 63 ETIs were recorded with a complete dataset by sixteen HEMS physicians. The FPSR significantly improved from the BASELINE (76.7%, 95% CI ±10.7) to the BOAH phase (95.1%, 95% CI ± 5.4, p=.004). Lowest postintubation oxygen saturation was 83.8% ± 16.1 SD (BASELINE) vs. 88.4% ± 13.5 SD (BOAH, p=0.014), and the duration of successful ETI attempts was 34 s ± 24.1 SD in the BASELINE phase and 32 s ± 14.9 SD in the BOAH phase (p=0.41).

**Conclusion:**

The combination of the BOAH airway bundle and FFC-based training was associated with a higher observed FPSR and improved oxygenation outcomes when it was used on real emergency patients by non-anaesthetist-staffed HEMS crews. These results suggest that skills acquired during cadaver-based training, which included practice of a comprehensive airway bundle, can be reliably transferred to real-life missions.

## Background

Endotracheal intubation (ETI) remains the gold standard for airway management [1]. Despite the availability of alternatives, this life-saving procedure must be mastered by all physicians in the prehospital setting, regardless of their board certification [1–3]. The probability of difficult airway situations is increased in the prehospital patient population [4, 5]. Predictive tools for difficult airway management can be safely applied in the prehospital setting [6]; however, every effort should be made to avoid “cannot intubate, cannot ventilate” scenarios, as the consequences for critically ill patients may be fatal [1, 7].

The key performance indicator of airway competence is the first-pass success rate (FPSR), defined as the rate of successful placement of a tracheal tube on the first laryngoscopy attempt, confirmed via capnography. Successful first attempts avoid complications such as airway trauma, hypotension, and hypoxia [8, 9].

The number of ETIs performed by providers is correlated with successful airway management. However, the published minimum requirements of 100–200 TIs can be challenging to achieve for emergency physicians who are not specially trained as anaesthetists. The challenge of how expertise in this procedure can be gained and maintained outside the operating room remains [10–13].

Several adjuncts to airway management effectively increase the FPSR. Videolaryngoscopy (VL) augments laryngeal visualisation and the FPSR in critically ill adults [3, 8, 9, 13–18]. Training with a Macintosh-style VL may not only improve FPSR with VL itself but may also translate into improved FPSR when direct laryngoscopy is performed [19, 20]. A meta-analysis of randomised controlled trials suggested that FPSR achieved with videolaryngoscopy may be lower in the prehospital setting than in the in-hospital setting (RR 0.75, 95% CI 0.57 to 0.99, *p* = 0.04, I^2^ = 95%) [21].

Additionally, the use of a bougie-first strategy significantly improves the FPSR by up to 14%, albeit training is needed [18, 22–24]. Patient positioning, such as ramping and head elevation, often referred to as “flextension” of the cervical spine, can greatly improve intubation conditions [24–29]. Selective extralaryngeal manipulation (ELM), such as bimanual laryngoscopy, may significantly improve laryngeal visualisation [30, 31].

However, procedures and devices alone do not improve outcomes; they must be conceptualised in a structured airway protocol and supported by training to ensure competence even under challenging prehospital circumstances [2, 23, 32].

Procedural training using manikins with or without medical simulation has been the standard for decades [32–34]. However, fresh frozen cadavers (FFC) are more realistic and superior for procedural skill acquisition [35–38]. For this study, we developed a comprehensive training course combining manikin training, medical simulation and FFC utilisation. The in vitro effects of the course have been previously validated [39]. At this point, it is unclear whether the in vitro effects of the bundle trained on FFCs can also be demonstrated in real-life prehospital intubations.

In contrast to training in the operating room, the cadaver lab offers the opportunity to deconstruct the airway management procedure into its smallest teachable unit with immediate mentoring and video feedback. This approach facilitates “deliberate practice”—a concept derived from high-performance sport—and enables trainees to make improvements while practising without relying on external feedback [40–43]. This combination of the best available evidence and a systematic training approach, aimed at providing the Best Of Airway management in HEMS (BOAH, HEMS: helicopter emergency medical service), is referred to as the BOAH course.

The first hypothesis of this study was that the FPSR would significantly increase under real-life conditions after the BOAH course was completed, reaching 90% without complications. This metric represents the proficiency in ETI. Second, we hypothesised that ETI using the well-trained VL and Bougie FIRST strategy (BOAH airway bundle) would not require more time than the standard techniques would, with process time serving as a surrogate for team training efficiency. Finally, we expected a decline in the proportion of patients with postintervention hypoxaemia (oxygen saturation < 90% within 30 min of intubation) as a measure of improved process quality [44, 45].

## Methods

### Study design

This study was designed as a prospective before-and-after cohort study with a quality improvement framework (BOAH initiative). The longitudinal framework consisted of four stages: “plan”, “do”, “check”, and “act” according to the Deming cycle [46].

During the “plan stage,” the potential for quality improvement was identified by prospectively evaluating 60 consecutive ETIs performed by the physicians of Cologne HEMS using only direct laryngoscopy without a bougie and improved positioning. These data are referred to as the BASELINE.

**Fig. 1:**
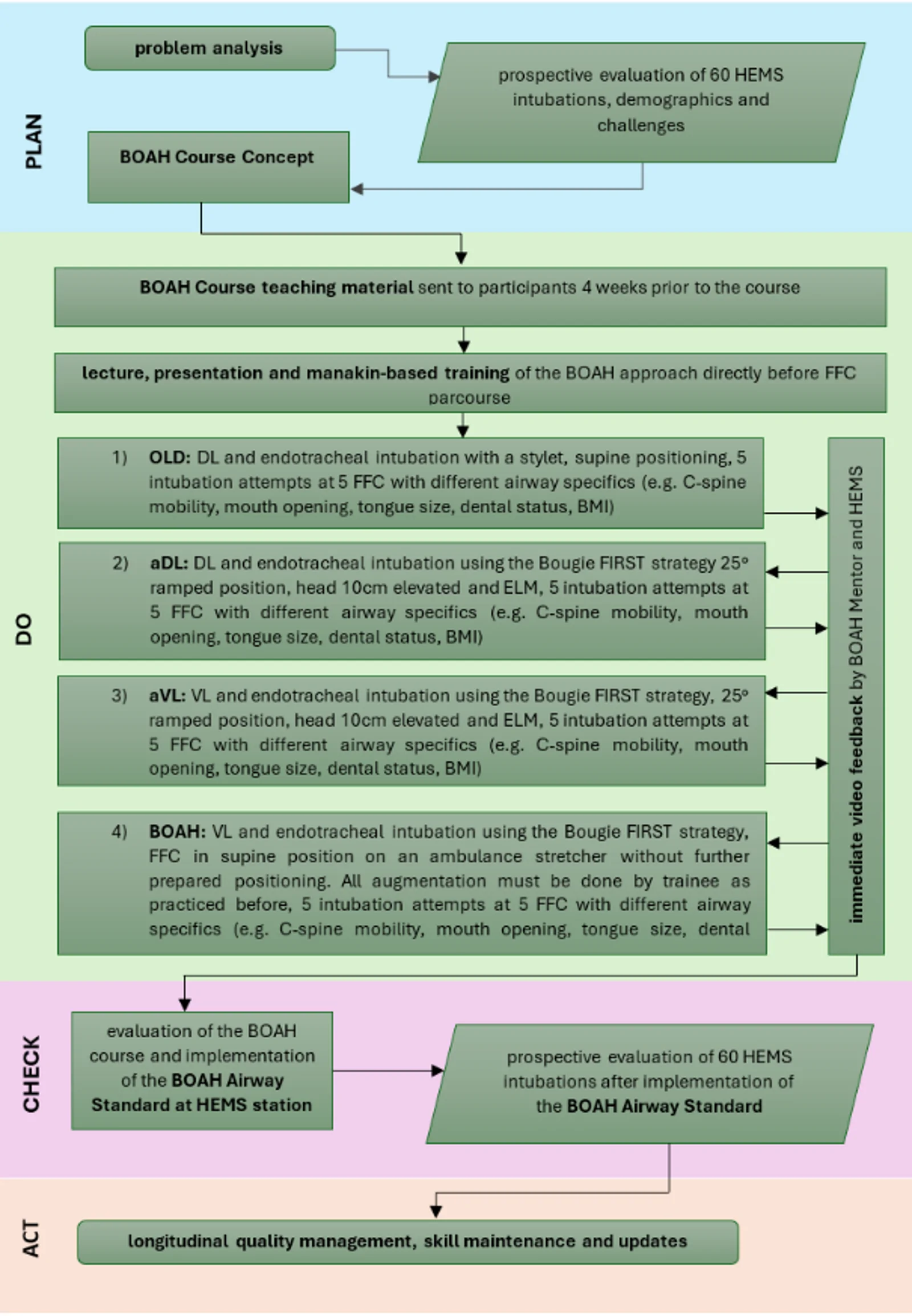
Flowchart of the BOAH airway project, outlining the planning, execution, and evaluation of the BOAH airway course and implementation of the BOAH airway standard at Christoph 3 HEMS station. HEMS=hospital emergency medical service or servicemember, BOAH=best of airway in HEMS, FFC=fresh frozen cadavers, OLD=airway standard Christoph 3 prior to BOAH project, aDL=augmented direct laryngoscopy, aVL=augmented videolaryngoscopy, BMI=body-mass-index

The educational intervention was drafted based on a comprehensive review of all the cases and a literature review (Fig. 1).

### Setting and participants

ETIs in this study were performed by 16 physicians from *Christoph 3* HEMS (Cologne HEMS) in Cologne, Germany. The Cologne metropolitan area is a densely populated economic region on the Rhine River with approximately 3.6 million residents. The region is a major transportation hub and is characterised by industry, several recreation areas with high activity of all sports, and heavy traffic.

The mandatory requirements of being a local HEMS physician are 6 months of critical care training, an 80-hour theoretical postgraduate course in prehospital emergency medicine, 50 supervised emergency missions, a 6-month full-time rotation as a prehospital emergency physician (≥ 300 independent missions), 2 weeks of paediatric anaesthesia training, completion of a critical care transport course and completion of the Advanced Trauma Life Support (ATLS^®^) course. Additionally, the annual continuing medical education requirement must be met. The physicians of Cologne HEMS are predominantly senior residents or faculty members of the Department of Traumatology and Orthopedic Surgery at Cologne-Merheim Medical Centre, Cologne, Germany.

**Fig. 2:**
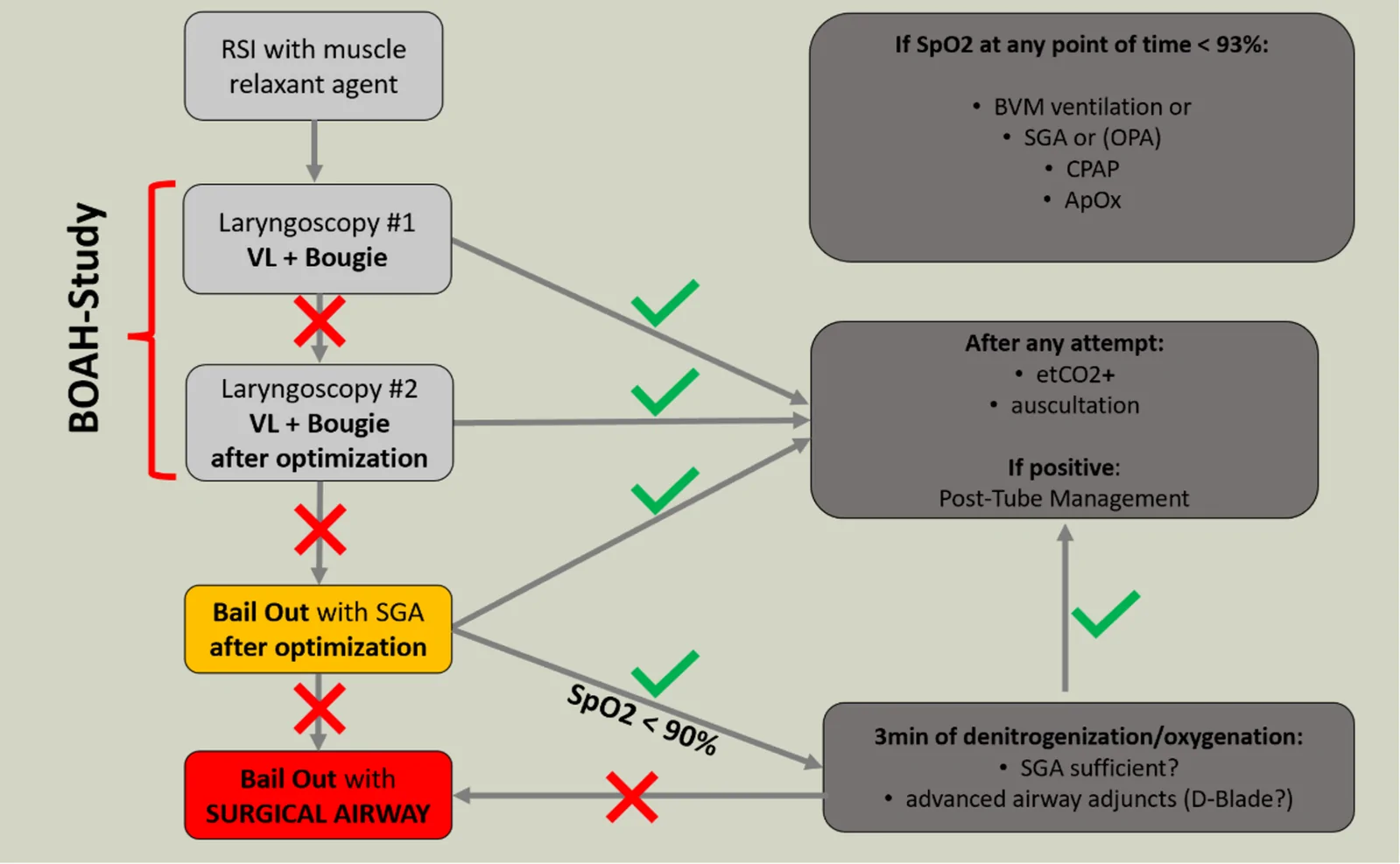
BOAH airway algorithm at Christoph 3 HEMS station. HEMS=hospital emergency medical service or servicemember, BOAH = best of airway in HEMS, RSI = rapid sequence induction, VL = videolaryngoscopy, SGA = supraglottic airway, OPA = oropharyngeal airway, CPAP = continuous positive airway pressure, ApOx = apneic oxygenation, SpO2 = oxygen saturation, etCO2 = end-tidal carbondioxide

The “do-stage” emphasised measures to maximise the FPSR. We performed a cadaver-based educational intervention of the BOAH airway bundle for all 16 physicians, consisting of an airway algorithm incorporating the VL, bougie-FIRST, and bail-out strategies (Fig. 2 shows the detailed airway algorithm at Cologne HEMS) [31].

In training and the BOAH phase, only the GlideScope^®^ Go™ (Verathon, Bothell, USA) VL with a size 4 MacIntosh-like blade and S-Guide^®^ 15Fr 65 cm bougie (VBM Medical, Sulz a.N., Germany) were used. No external equipment was used on scene in the BOAH phase. The five FFCs were selected and modified to reflect the airway characteristics of the prehospital patient cohort of the BASELINE phase. During the intervention, each participant was supervised by a BOAH mentor and supported by a flight paramedic who regularly served as a technical crew member at the local HEMS (HEMS-TC). Detailed in vitro results from the BOAH course have been previously published [35].

During the “check-stage”, after the FFC-based training of the BOAH airway bundle (VL and bougie FIRST strategy) and implementation of the aforementioned devices, another 60 consecutive prehospital ETIs were prospectively evaluated at Cologne HEMS without refresher sessions. All ETIs performed primarily by study participants (physicians of Cologne HEMS) on adult patients (18 years of age or older) were eligible for inclusion in the study.

In these cases, all the techniques trained in the intervention were used by the same physicians who performed in the BASELINE phase to evaluate the effectiveness of the airway bundle in real-life situations (BOAH group) and reduce the risk of bias. This stage lasted from September 2022 to June 2023. The primary outcome measure was the FPSR without complications (death, cardiac arrest, i.e., chest compressions, any new airway trauma, and oesophageal intubation). Secondary outcome measures included the lowest post intubation oxygen saturation within 30 min of intubation (SpO_2_, measured using fingertip oximetry), total number of ETI attempts, duration of successful ETI attempts (recorded by HEMS-TC using a stopwatch), post ETI end-tidal carbon dioxide (etCO_2_), subjective conditions of the airway at ETI, and procedural data (use of VL, a bougie, suction, ELM, muscle relaxant agents, or bail-out strategies). Demographic data, airway characteristics, and clinical parameters were documented for group comparisons on a study-specific data sheet after each mission, in accordance with the four-eyes principle, by the physician and the HEMS-TC.

The “act-stage” involving measures to ensure sustainability is not within the scope of the present analysis.

### Data collection and statistical methods

Data were collected using a predefined standardised documentation form by the intubating physician during and immediately after the mission. ETIs were timed in a standardised manner with a stopwatch by the HEMS-TC. The physician had already performed the procedure in the same manner during FFC training.

The main author performed longitudinal dataset quality assurance to ensure a high degree of data completeness. The data were transferred to a spreadsheet (Microsoft Excel version 365; Redmond, WA, USA). Missing data were not replaced.

Statistical analyses were performed using SPSS statistical software (version 28; IBM Inc., Armonk, NY, USA). Categorical variables are presented as absolute and relative values, and continuous variables are presented as the means with standard deviations (SD) and 95% confidence interval (95% CI). Group comparisons were performed using the chi-square test for categorical variables and the Mann–Whitney U test for continuous variables. Data distribution testing was performed before using the Mann–Whitney U test.

The sample size was calculated a priori, assuming a medium effect size for the primary outcome of the FPSR (increase of > 10%) and a planned statistical power of 0.8 (G^*^Power 3.1.9.7).

A sensitivity analysis was conducted to assess the potential impact of missing SpO_2_ and etCO_2_ values.

Statistical significance was set at *p* < 0.05.

### Study approval

This study was registered in the German Registry of Clinical Trials (DRKS00023889 on April 1st, 2021; https://www.drks.de). Ethical approval was granted prior to the initiation of the study by the Ethics Committee of the Faculty of Medicine, University of Witten/Herdecke, Germany (No. 86/2019).

## Results

The same sixteen physicians participated and performed all intubations (ETIs) in the BASELINE and BOAH phases (mean age 38, 15 males, 10 consultants, and 6 senior residents). In the BASELINE phase 60 of 64 consecutive, primary ETIs were recorded; in the BOAH phase, 61 of 63 were recorded. The reason for exclusion was always a lack of study forms.

Patient demographics and clinical data for the BASELINE and BOAH phases are summarised in Table 1.

**Table 1:** Patient demographics and clinical data of BASELINE and BOAH

Parameter	BASELINE (*n* = 60)	BOAH (*n* = 61)		*p*-value
**Patient Age at ETI [mean ± SD]**	62.8 ± 20.0	58.6 ± 19.4		0.20*
**Gender Patient Male, n [%]** **Patient Body Length [cm; mean ± SD]** **Patient Body Weight [kg; mean ± SD]**	43 [71.7] 180 [± 8] 84.2 [± 14.5]	41 [67.2] 177 [± 6] 86.5 [± 15.5]		0.28** 0.83 0.10*
**BMI [kg/m²; mean ± SD]**	23.1 [± 4.8]	27.7 [± 4.7]		0.09*
**Indication for HEMS ETI**				
RESP n [%]	1 [1.7]	4 [6.6]		0.19**
NEURO, n [%]	14 [23.3]	22 [36.1]		0.22**
TRAUMA B respiratory insufficiency, n [%] TRAUMA C hemodynamic instability, n [%] TRAUMA D traumatic brain injury, n [%]	1 [1.7] 5 [8.3] 15 [25.0]	13 [21.3] 5 [8.2] 7 [11.5]		< 0.001** 0.62** 0.04**
CPR, n [%] OTHER, n [%]	27 [45.5] 4 [6.7]	24 [39.3] 1 [1.6]		0.33** 0.18**
**Setting of ETI**				
Ambulance, n [%]	35 [58.3]	30 [49.2]		0.20**
Home/Bed/Floor, n [%] Outdoor, n [%]	20 [33.3] 4 [6.7]	16 [26.2] 15 [24.6]		0.26** 0.01**
**Airway characteristics** Cormack-Lehane grade [median] Thyromental distance [cm; mean ± SD] Mallampati [median] Mouth opening [cm; mean ± SD] Beard, n [%] Maxillofacial trauma, n [%] Macroglossia, n [%] Signs of airway obstruction, n [%] Dental pathologies, n [%] Limited c-spine reclination, n [%] In-line immobilization, n [%]	2 6.63 [± 1.34] 2 3.64 [± 0.93] 6 [10] 8 [13.3] 4 [6.7] 5 [8.3] 21 [35] 23 [38.3] 23 [38.3]	2 6.84 [± 1.27} 2 4.36 [± 1.86] 11 [18.0] 14 [23.0] 5 [8.2] 4 [6.6] 18 [29.5] 25 [41.0] 23 [37.7]		0.49** 0.05* 0.52** 0.02* 0.16** 0.13** 0.74** 0.74** 0.33** 0.46** 0.55**

BOAH = Best-of Airway in Helicopter Emergency Medical Service; RESP = respiratory insufficiency defined as respiratory rate > 29/min or < 6/min or hypoxia or failure of non-invasive ventilation; NEURO = neurological emergency; unconsciousness or altered mental status with aspiration risk without traumatic brain injury; TRAUMA B = Intubation for respiratory insufficiency after trauma, multisystem trauma with SpO_2_ (peripheral oxygen saturation) < 90% with supplemental oxygen; TRAUMA C = Intubation for hemodynamic instability after trauma, blood pressure < 90mmHg; TRAUMA D = Intubation for traumatic brain injury Glasgow Coma Scale 3—8; CPR = Intubation during or after cardio-pulmonal resuscitation; OTHER = Other indications like pain control or rescue tactics; ETI = endotracheal intubation; * Mann-Whitney-U-Test; ** Chi-Square-Test

The groups did not differ significantly in terms of age, sex, body length, weight, or body mass index (BMI). Similarly, no significant differences were found in airway characteristics such as the Cormack–Lehane grade, Mallampati score, facial hair, maxillofacial trauma, airway obstruction signs, dental pathologies, limited c-spine reclination, or the need for in-line immobilisation. However, thyromental distance and mouth opening significantly differed between the groups.

The most common indications for ETI were cardiopulmonary resuscitation (CPR), followed by trauma, neurological emergencies, and respiratory insufficiency.

Most ETIs were performed on stretchers in the ambulance vehicle. Others were performed at the patient’s home, in bed, or on the floor. A smaller proportion of patients were intubated outdoors: 6.7% in the BASELINE cohort and 24.6% in the BOAH cohort (*p* < 0.001).

In terms of the first hypothesis, the FPSR increased significantly from 76.7% (95% CI in the BASELINE cohort to 95.1% in the BOAH cohort (95% CI ± 5.4, *p* = 0.004; Table 2).

**Table 2:** Clinical and procedural outcome data pre and post BOAH bundle implementation

Clinical, Procedural and processual outcome data				
**Parameter**	**BASELINE** (*n* = 60)	**BOAH** (*n* = 61)		**p-value**
***Primary Outcome Measure***: **FPSR**,** n [%; 95% CI]**	46 [76.7 ± 10.7]	58 [95.1 ± 5.4]		0.004**
***Secondary Outcome Measures***: **Number of ETI attempts** 2 Attempts, n 3 Attempts, n 4 Attempts, n **Duration of successful ETI attempt** **[s; mean; ± SD]** **[s; mean; ± 95% CI]** **Post ETI SpO**_**2**_ **[%; mean ± SD]** **[%; mean; ± 95% CI]** **Post ETI hypoxaemia [SpO**_**2**_ **< 90%]** **Critical ETI hypoxaemia [SpO**_**2**_ **< 80%]** **Post ETI etCO**_**2**_ **[mmHg**,** mean ± SD]** **[mmHg**,** mean ± 95% CI]** **Subjective conditions of the airway at ETI** Very good, n [%] Good, n [%] Moderate, n [%] Poor, n [%]	12 1 1 3 [± 24.1] 34 [± 6.3] 83.8 [± 16.1] 83.8 [± 4.1] 27 [45.0%] 9 [15.0%] 32.9 [± 12.4] 32.9 [± 3.2] 11 [18.3] 17 [28.3] 19 [31.7] 9 [15.0]	3 0 0 32 [± 14.9] 32 [± 3.8] 88.4 [± 13.5] 88.4 [± 3.5] 15 [24.6%] 7 [11.5%] 35.9 [± 10.8] 35.9 [± 2.8] 22 [36.1] 23 [37.7] 7 [11.5] 4 [6.6]		0.41* 0.014* 0.33* 0.002*
**Procedural Data of ETI**				
Use of VL, n [%] Use of bougie, n [%] Use of suction, n [%] Use of ELM, n [%] Use of BAIL OUT, n [%] Use of EFONA, n [%] Use of Flextension, n [%]	4 [6.7] 3 [5.0] 14 [23.3] 22 [36.7] 1 [1.7] 0 [0] ND	61 [100.0] 59 [96.7] 11 [18.0] 16 [26.2] 0 [0] 0 [0] 52 [85.2]		< 0.001** < 0.001** 0.49** 0.24**
	**BASELINE without CPR** (*n* = 33)	**BOAH without CPR** (*n* = 37)		
**Anaesthesia Data**				
Use a muscle relaxant agent, n [% of patients, CPR excluded] Use of analgesics and sedatives, n [% of patients, CPR excluded]	25 [75.8] 33 [100]	30 [81.1] 37 [100]		0.38**

BOAH = Best-of Airway in Helicopter Emergency Medical Service; FPS = First-pass success; ETI = endotracheal intubation; ELM = extra laryngeal manipulation, CPR = cardiopulmonary resuscitation; BAIL OUT = use of bail out airway strategy like laryngeal mask, laryngeal tube or CRICeFONA = cricothyroidotomy, ND = not documented; * Mann–Whitney-U Test; ** Chi-Square Test

In the BASELINE cohort, more than one intubation attempt was required in 14 cases (meeting the ATOM Core Outcome Set definition of difficult ETI), with three attempts required in one case and four attempts required in another case. In one patient, a laryngeal mask was used as a bail-out strategy. Conversely, in the BOAH cohort, a second attempt was required in three cases (difficult ETI), and no intubation required more than two attempts or bailout procedures. A front-of-neck airway procedure was not performed in either cohort.

To confirm the secondary hypothesis, we measured the duration of each successful attempt. There were no significant differences in the group comparisons (34 s 95% CI ± 6.3 BASELINE group and 32 s 95% CI ± 3.8 BOAH group, *p* = 0.41).

There were no missing data for the FPSR or the duration of the successful ETI attempt.

To verify the third hypothesis, we compared the proportions of patients with postintubation critical hypoxaemia (Table 2). While only powered for the FPSR, the group comparison revealed a significant increase in oxygen saturation above 90% SpO_2_ (BASELINE group 55.0% vs. BOAH group 75.4%). SpO_2_ follow-up data over 30 min were missing for seven (11.6%, BASELINE group) and nine (14.8%, BOAH group) patients.

Postintubation etCO_2_ values were comparable between the cohorts (32.9 mmHg 95% CI ± 3.2 vs. 35.9 mmHg 95% CI ± 2.8; *p* = 0.33). The etCO_2_ values were missing for four (6.6%, BASELINE group) and one (1.6%, BOAH group) patient.

Providers subjectively rated the airway condition at ETI on a four-point scale. The operator-reported airway condition ratings of the BOAH cohort were significantly better. The potential for reporting bias inherent to subjective operator-reported outcomes should be acknowledged.

Airway trauma due to the procedure was reported in 11 BASELINE (18.3%) and 2 BOAH (3.3%) patients. Airway trauma was defined on the study data form as any airway injury, including mucosal bleeding and dental injury, identified by the operator during or following the intubation procedure. Pulmonary complications (clinical pulmonary aspiration) were documented in seven and three patients (11.7% BASELINE group vs. 4.9% BOAH group).

The VL use rate was 6.7% in the BASELINE group compared with 100% in the BOAH group; a “bougie first approach” was used in 5.0% vs. 96.7% of all cases (*p*<0.001), respectively, representing high protocol compliance.

Among non-CPR patients, muscle relaxants were used in 75.8% (BASELINE group) and 81.1% (BOAH group) of cases (*p* = 0.38). No further conclusions on the adequacy of the dosage and quality of the performed anaesthesia were made (100% use of sedatives and analgesics in non-CPR cases in the BOAH group).

## Discussion

In this prospective study, the FPSR without complications in real-life HEMS settings improved by 18.4% (from 76.7% in the BASELINE to 95.1% in the BOAH) in a single German non-anaesthetist-staffed HEMS through the implementation of an evidence-based airway protocol in conjunction with FFC-based training. No more than two intubation attempts were performed in the BOAH group. As Bernhard et al. reported, “The first shot is often the best shot”.^13^ The BOAH bundle focuses on maximising the chance for success in the first intubation attempt. Therefore, an escalating airway strategy, such as the more generalised vortex approach, is not used [47].

The FPSR, which represents procedural competence prior to the intervention, was regarded as suboptimal for HEMSs (BASELINE 76.7%); however, it is likely a realistic reflection of the standard of care rather than a negative outlier. In a meta-analysis, Park et al. reported an overall FPSR of 84.1% across 42,081 airway cases in 83 emergency departments in 10 countries. The “trauma-only” subgroup had an FPSR of 81.8% [48].

Hossfeld et al. reported an FPSR of 90.0% since the introduction of VL in 2009 at another German HEMS (Christoph 22 in Ulm). The emergency physicians of the Ulm HEMS are all consultant-level anaesthesiologists [3]. A Dutch study reported an FPSR of 85.9% with direct laryngoscopy and 86.9% with VL among HEMS physicians [49]. A multicentre observational study of all 12 Swiss HEMSs reported an FPSR of 87.6% [50]. Sydney HEMS, internationally renowned for its training and pursuit of clinical excellence, published an FPSR of 97% in 2022 [51].

Confounding factors, such as patient demographics, body weight, and intubation indications, did not differ significantly between the two groups. Although airway characteristics were generally comparable, they differed in some aspects. Significantly more patients in BOAH were intubated outdoors and presented with traumatic injuries associated with respiratory insufficiency. Thyromental distance and mouth opening also differed significantly between the groups. Notably, the difference in mouth opening (> 0.5 cm in BOAH) may be clinically relevant. Overall, these baseline differences may have contributed to the observed differences in FPSR.

According to the recommended definition of intubation time by the American National Association of EMS Physicians (time interval from the laryngoscope entering the mouth until tube placement) [52], the intubation time was shorter in the BOAH group, but the difference was not statistically significant. This is noteworthy, since an airway procedure using VL and a bougie-first strategy includes the additional step of the “railroading” of the endotracheal tube over the bougie. In addition to the narrower standard deviations in intubation time, this observation might reflect the team training aspect of the BOAH course. Caputo et al. reported that desaturation to an SpO_2_ < 90% rarely occurred for intubations lasting less than 60 s if the patient was preoxygenated and denitrogenated adequately prior to the intubation attempt [53]. Accordingly, the postintubation SpO_2_ was significantly higher in the BOAH group (75.0% of all cases). End tidal capnography after ETI is mandatory in the BOAH airway protocol to control endotracheal tube positioning. There was a trend towards higher etCO_2_ values in the BOAH group, and etCO_2_ values can also reflect haemodynamics [54]. Further haemodynamic parameters, such as heart rate and blood pressure, were not documented before or after intubation in the study.

HEMS physicians subjectively reported that airway conditions were significantly better in the BOAH group. Objective parameters defining a difficult airway, such as BMI, Cormack–Lehane grades, thyromental distance, Mallampati scores, mouth opening, facial hair, maxillofacial trauma, airway obstruction, dental pathologies and limited reclination did not significantly differ between the BASELINE and BOAH groups. Providers were trained to accept the “minimal intubatable view” instead of losing time in pursuit of optimisation of the intubation view, a concept that has value in patients with traumatic brain injuries and a high likelihood of cervical spine injuries [55, 56]. The minimal view was defined as the deliberate placement of the bougie at the earliest point at which safe insertion appeared visually feasible, without additional attempts to maximise glottic exposure.

Overall, protocol adherence was high. VL, the bougie-first strategy, and the flextension technique for head positioning were documented as key procedural pearls of the BOAH course in nearly all patients in the BOAH group. ELM and suction were used less frequently. These procedures can be helpful for selected patients; hence, they should be taught and mastered. In particular, suction-assisted laryngoscopy and airway decontamination (SALAD) is an airway technique of great value and requires some training and simulation prior to utilisation in the field [57, 58]. Mastering airway decontamination methods is mandatory because gastric content, which blocks airways, is the most common reason for failed first attempts using a VL and bougie-first strategy [59]. Three cuff ruptures were recognised as technical incidents in this study, one in the BASELINE group and two in the BOAH group. No evidence was found that the VL or bougie technique was associated with increased endotracheal tube rupture rates. With the bougie at hand and being proficient in its use, this complication can readily be managed by reintroducing the bougie and exchanging the endotracheal tube using the Seldinger technique under VL.

There is evidence for the impact of team training on the FPSR from simulation [60], and high-performing prehospital teams [51, 58]. Therefore, the BOAH course emphasised the importance of team training and empowerment of the HEMS-TC by repeatedly changing roles during training and adapting the position title to “operator 1” (performing VL) and “operator 2” (offering assistance and external feedback), which accounts for the values at both positions.

The strength of this study is its longitudinal design, which translated a real-life HEMS problem into an FFC training model, controlling the success of bundle training in real-life HEMS settings. However, because a comprehensive intervention bundle was implemented, the observed improvements cannot be attributed exclusively to the FFC training or to any single intervention. In addition, the interprofessional approach, which encouraged the HEMS-TC to play a more active role in prehospital endotracheal intubation, contributes to its strength.

### Study limitations

The findings of this study should be interpreted in the context of the highly organized HEMS system in which it was conducted. Standardized equipment, physician experience, and structured airway training may have contributed to the observed results. Consequently, the generalizability of these findings to EMS systems with different staffing models, airway equipment, levels of provider experience, or training opportunities may be limited. However, the equipment standards and training concept are described in sufficient detail in this study to facilitate adaptation by other EMS systems. Access to FFC itself remains a limiting resource that may restrict broader implementation.

Intervention bias might have contributed to the observed increase in the FPSR in this study. The BOAH airway bundle and course address several reasons for difficulties in adopting new concepts, such as inadequate guidelines, lack of training, and unsuitable equipment; however, the sustainability of this intervention beyond the study setting should be demonstrated [61, 62]. As a major limitation, this study involved the implementation and training of a complex bundle of interventions, and determining the effects of specific elements—such as the use of VL, the Bougie FIRST strategy, or FFC-based training of the airway plan—on the improvement of the FPSR is not possible. Moreover, the use of VL and bougies has become significantly more widespread in clinical practice since the study began, which may have further increased providers’ familiarity with those tools. The goal of 100% use of muscle relaxants in non-CPR cases in the BOAH phase was clearly not met, regardless of whether it was a protocol recommendation. While there was no significant difference in muscle relaxant use between the groups, a relevant effect on the FPSR in both groups cannot be ruled out. Muscle relaxants are an essential clinical component for a higher FPSR.

The study did not include certain essential physiological or procedural data—such as preoxygenation methods, apnoeic oxygenation techniques, or haemodynamic readings—that can influence oxygen saturation after intubation. Since the power calculation was based on the primary outcome of an increased FPSR, conclusions regarding secondary outcomes such as the hypoxia rate cannot be drawn with the same degree of certainty. Data collection in real-life HEMS missions without additional staff may affect data quality.

Because outcomes such as airway trauma were operator-reported, there is a potential risk of observer and reporting bias. Nevertheless, all cases were documented using a standardized protocol, which likely improved the consistency of outcome reporting.

## Conclusion

In conclusion, prehospital airway management could be performed more safely and successfully by providers without exposure to elective anaesthesia on a daily basis if emphasis is placed on airway protocols and evidence-based procedures such as VL, bougie-first strategies, patient positioning, and high-quality teaching using, for example, FFCs, team training, and rapid feedback loops, enabling deliberate practice and single-technique mastery. In this study, FFC-augmented airway management training translated into reproducible real-life outcomes and may therefore be considered a cost- and time-efficient option for emergency medical services. It should be noted that the observed effects cannot be attributed solely to FFC due to concurrent interventions of the study. The well-documented and published concepts of the BOAH initiative add some data on the relationship between competence in tracheal intubations in simulation and competence in tracheal intubations in real-life situations.

## Data Availability

The data that support the findings of this study were collected by the BOAH study group. The data are not publicly available due to restrictions imposed in part by the local ethics committee. The data are available from the corresponding author upon reasonable request and with permission from the BOAH study group.

## Declarations

### Study registry

German Clinical Studies Registry (DKRS, Study-ID 00023889, registered 01 April 2021, https://www.drks.de).
